# Ten Simple Rules for a Successful Collaboration

**DOI:** 10.1371/journal.pcbi.0030044

**Published:** 2007-03-30

**Authors:** Quentin Vicens, Philip E Bourne

Scientific research has always been a collaborative undertaking, and this is particularly true today. For example, between 1981 and 2001, the average number of coauthors on a paper for the Proceedings of the National Academy of Sciences U S A rose from 3.9 to 8.4 [1]. Why the increase? Biology has always been considered the study of living systems; many of us now think of it as the study of complex systems. Understanding this complexity requires experts in many different domains. In short, these days success in being a biologist depends more on one's ability to collaborate than ever before. The Medical Research Centers in the United Kingdom figured this out long ago, and the new Janelia Farm research campus of the Howard Hughes Medical Institute in the United States has got the idea, as it strongly promotes intra- and inter-institutional collaborations [2].

Given that collaboration is crucial, how do you go about picking the right collaborators, and how can you best make the collaboration work? Here are ten simple rules based on our experience that we hope will help. Additional suggestions can be found in the references [3,4]. Above all, keep in mind that these rules are for both you and your collaborators. Always remember to treat your collaborators as you would want to be treated yourself—empathy is key.

## Rule 1: Do Not Be Lured into Just Any Collaboration

Learn to say no, even if it is to an attractive grant that would involve significant amounts of money and/or if it is a collaboration with someone more established and well-known. It is easier to say no at the beginning—the longer an ill-fated collaboration drags on, the harder it is to sever, and the worse it will be in the end. Enter a collaboration because of a shared passion for the science, not just because you think getting that grant or working with this person would look good on your curriculum vitae. Attending meetings is a perfect opportunity to interact with people who have shared interests [5]. Take time to consider all aspects of the potential collaboration. Ask yourself, will this collaboration really make a difference in my research? Does this grant constitute a valid motivation to seek out that collaboration? Do I have the expertise required to tackle the proposed tasks? What priority will this teamwork have for me? Will I be able to deliver on time? If the answer is no for even one of these questions, the collaboration could be ill-fated.

Enter a collaboration because of a shared passion for the science . . .

## Rule 2: Decide at the Beginning Who Will Work on What Tasks

Carefully establishing the purpose of the collaboration and delegating responsibilities is priceless. Often the collaboration will be defined by a grant. In that case, revisit the specific aims regularly and be sure the respective responsibilities are being met. Otherwise, consider writing a memo of understanding, or, if that is too formal, at least an e-mail about who is responsible for what. Given the delegation of tasks, discuss expectations for authorship early in the work. Having said that, leave room for evolution over the course of the collaboration. New ideas will arise. Have a mutual understanding up-front such that these ideas can be embraced as an extension of the original collaboration. Discuss adjustments to the timelines and the order of authors on the final published paper, accordingly. In any case, be comfortable with the anticipated credit you will get from the work. The history of science is littered with stories of unacknowledged contributions.

## Rule 3: Stick to Your Tasks

Scientific research is such that every answered question begs a number of new questions to be answered. Do not digress into these new questions without first discussing them with your collaborators. Do not change your initial plans without discussing the change with your collaborators. Thinking they will be pleased with your new approach or innovation is often misplaced and can lead to conflict.

## Rule 4: Be Open and Honest

Share data, protocols, materials, etc., and make papers accessible prior to publication. Remain available. A trusting relationship is important for the collaborative understanding of the problem being tackled and for the subsequent joint thinking throughout the evolution of the collaboration.

## Rule 5: Feel Respect, Get Respect

If you do not have respect for the scientific work of your collaborators, you should definitely not be collaborating. Respect here especially means playing by Rules 2–4. If you do not respect your collaborators, it will show. Likewise, if they don't respect you. Look for the signs. The signs will depend on the personality of your collaborators and range from being aggressive to being passive–aggressive. For example, getting your tasks done in a timely manner should be your priority. There is nothing more frustrating for your collaborators than to have to throttle their progress while they are waiting for you to send them your data. Showing respect would be to inform your collaborator when you cannot make a previously agreed-upon deadline, so that other arrangements can be made.

## Rule 6: Communicate, Communicate, and Communicate

Consistent communication with your collaborators is the best way to make sure the partnership is going in the planned direction. Nothing new here, it is the same as for friendship and marriage. Communication is always better face-to-face if possible, for example by traveling to meet your collaborators, or by scheduling discussion related to your collaborations during conferences that the people involved will attend. Synchronous communication by telephone or video teleconferencing is preferred over asynchronous collaboration by e-mail (data could be exchanged by e-mail prior to a call so that everyone can refer to the data while talking).

## Rule 7: Protect Yourself from a Collaboration That Turns Sour

The excitement of a new collaboration can often quickly dissipate as the first hurdles to any new project appear. The direct consequence can be a progressive lack of interest and focus to get the job done. To avoid the subsequent frustrations and resentment that could even impact your work in general, give three chances to your collaborators to get back on track. After all, your collaborators could just be having a difficult time for reasons outside of their control and unanticipated at the time the collaboration started. After three chances, if it feels like the collaboration cannot be saved, move on. At that point try to minimize the role of your collaborators in your work: think carefully about the most basic help you need from them and get it while you can (e.g., when having a phone call or a meeting in person). You may still need to deal with the co-authorship, but hopefully for one paper only!

## Rule 8: Always Acknowledge and Cite Your Collaborators

This applies as soon as you mention preliminary results. Be clear on who undertook what aspect of the work being reported. Additionally, citing your collaborators can reveal your dynamism and your skills at developing prosperous professional relationships. This skill will be valued by your peers throughout your career.

## Rule 9: Seek Advice from Experienced Scientists

Even though you may not encounter severe difficulties that would result in the failure of the partnership, each collaboration will come with a particular set of challenges. To overcome these obstacles, interact with colleagues not involved in the work, such as your former advisors or professors in your department who have probably been through all kinds of collaborations. They will offer insightful advice that will help you move beyond the current crisis. Remember, however, that a crisis can occasionally lead to a breakthrough. Do not, therefore, give up on the collaboration too easily.

## Rule 10: If Your Collaboration Satisfies You, Keep It Going

Ever wondered why a pair of authors has published so many papers together? Well, it is like any good recipe: when you find one that works, you cook it again and again. Successful teamwork will tend to keep flourishing—the first paper will stimulate deeper and/or broader studies that will in turn lead to more papers. As you get to know your collaborators, you begin to understand work habits, strengths but also weaknesses, as well as respective areas of knowledge. Accepting these things and working together can make the work advance rapidly, but do not hurry: it takes time and effort from both sides to get to this point.

Collaborations often come unexpectedly, just like this one. One of us (PEB) as Editor-in-Chief was approached not just with the idea for these Ten Rules, but with a draft set of rules that needed only minor reworking. As you can see, we have obeyed Rule 8. 
